# The Burden of Not Doing

**DOI:** 10.3390/pediatric16020028

**Published:** 2024-04-25

**Authors:** Maurizio Aricò

**Affiliations:** Pediatrics, Hospital of Pescara, 65124 Pescara, Italy; maurizio.arico@asl.pe.it

The cultural and professional growth of a physician is a long process, spanning over more than 10 years. This period primarily involves the acquisition of technical skills, enabling future/young medical doctors to know what to do and “how to do it”. This means learning about physiology and pathophysiology as a preliminary step in understanding and recognizing the main features of innumerous human diseases, with the aim of caring for all and hopefully curing the majority of patients [1].

Comprehending new diagnostic procedures at an accelerating pace, which all too often accrue alongside traditional techniques rather than replacing them, is a daily challenge, not only for apprentices but also, and maybe even more so, for seasoned physicians.

In the past, doctors were considered almost “oracle”-like figures, the owners of scientific knowledge, and thus the masters of defeating disease. However, over the last five to six decades, this relationship has deeply changed, in part for good reason. Patients are now on average much better informed, and patient empowerment has been a positive development in the field of medicine. 

In our daily work, we constantly face people seeking reassurance wherever possible and people diagnosed with newly diagnosed and unwelcome diseases. However, it is undeniable that the glorious role of the “family doctor” has substantially melted away. The sheer volume of medical knowledge stemming from developments in scientific research and medical technology overruns the purview of a single professional. Continuous medical education (CME) is a noble and useful attempt, but we can all acknowledge that it falls short of a return to the past. Thus, the image of a “general clinician” who is able to wade through a sea of medical knowledge is now long-lost, replaced by the idea of “super-specialist docs”. Patient expectations are now that super-specialists will be able to fix (almost) everything quickly and easily. Thus, if fever (especially in children) does not go away, if pain is not relieved, and if disease is not essentially cured in an instant, the perception is that something in the health service has malfunctioned. The immediate expectation is that you, as a super-specialist, are a kind of “magician” who can always conjure a definitive diagnostic procedure or a cutting-edge (usually expensive) wonder drug appropriate for the problem, and it will be solved.

In this scenario, are we allowed to “not do”? How often do we anticipate that a viral infection will go away in due course and yet still prescribe an antiviral or an antibiotic so that it does not appear that we are poorly informed? By this logic, there is no need for lengthy explanations to parents, and we play it safe regarding potential legal issues. Of course, writing is faster than talking, and indeed, the time permitted for listening, explaining, and discussing patient options has become increasingly short. The time in which a visit must be completed is codified by insurance or by clinic managers (twenty minutes max, please…). Breaking these codes may be regarded “old-fashioned” or simply unacceptable. Run and be “productive”. Prescribe antibiotics even for a flu-like fever: it is faster than explaining why it would be better not to. Who cares about “antimicrobial stewardship” in real life? Time is running out in the clinic today, whereas multi-resistant organisms are tomorrow’s concern [sorry, but nowadays, this is not true [2]].

In conclusion, how charismatic and committed must you be to afford the burden of “not doing”? 
